# Evaluating the implementation of active transportation infrastructure during the pandemic: a RE-AIM study

**DOI:** 10.1186/s12889-025-25432-y

**Published:** 2025-12-20

**Authors:** Zarah Monfaredi, Raktim Mitra, Valorie A. Crooks, Kevin Manaugh, Paul M. Hess, Meghan Winters

**Affiliations:** 1 Faculty of Health Sciences, Simon Fraser University, Blusson Hall Rm 11522, 8888 University Drive, Burnaby, BC V5A 1S6 Canada; 2 School of Urban and Regional Planning, Toronto Metropolitan University, 350 Victoria Street, Toronto, ON M5B 2K Canada; 3 Department of Geography, Simon Fraser University, 8888 University Dr W, Burnaby, BC V5A 1S6 Canada; 4 Department of Geography and Bieler School of Environment, McGill University, 805 Sherbrooke West, Montreal, QC H3A 0B9 Canada; 5 Department of Geography & Planning, University of Toronto, Toronto, ON Canada

**Keywords:** Implementation science, RE-AIM, Mixed Methods, Population health intervention, COVID-19, Street reallocations, Active transportation

## Abstract

**Background:**

The impact of urban transportation on human health is a critical area of study in public health. The COVID-19 pandemic created a need to re-imagine urban public spaces and trial built environment interventions under expedited timelines and physical distancing requirements. By expanding street space for active transportation during the pandemic, cities intended to enable access to essential destinations through ‘street reallocation’ interventions, with potential implications on physical and mental health. Using the RE-AIM framework, our aim is to evaluate the COVID-19 pandemic street reallocations in the cities of Vancouver, Toronto, and Montréal in Canada.

**Methods:**

Using a mixed methods approach, we evaluated street reallocation implementation across RE-AIM dimensions (Reach, Effectiveness, Adoption, Implementation, and Maintenance). We conducted a socio-spatial analysis using publicly available data, including cycling infrastructure, area-level sociodemographic characteristics, and measures of accessibility to destinations. To identify barriers and enablers of street reallocation implementation and to add nuance to the socio-spatial analysis, we conducted semi-structured interviews with municipal practitioners in 2022. We coded and analyzed the data using framework analysis.

*Reach*: In Vancouver, Toronto, and Montréal, 39.9%, 21.0%, and 32.9% of the population had access to street reallocations in their census tract. *Effectiveness*: In Vancouver, most street reallocations were in areas with high accessibility to (i.e., spatial proximity to) employment, and in Toronto and Montréal in areas with high accessibility to parks. *Adoption*: The majority of street reallocations were implemented near pre-existing cycling infrastructure. Though not targeted as users of the street reallocations, local business owners were important in the decision-making process. *Implementation*: A facilitator of street reallocation implementation was communication between municipal practitioners and community members, and continuous feedback cycles. *Maintenance*: Temporary materials used to initially construct street reallocations were high maintenance which resulted in high costs. Sustainability of street reallocation interventions hinges on community support.

**Conclusions:**

Our findings help to inform the work of municipal practitioners, public health professionals, and researchers looking to build healthier and more equitable transportation systems. A focus of future implementation science research may be on developing consistent metrics for built environment intervention evaluation.

**Supplementary Information:**

The online version contains supplementary material available at 10.1186/s12889-025-25432-y.

## Background

The potential influence of urban environments on human health has long been a concern amongst urban planners and public health practitioners [1–4]. As cities grapple with rapid urbanization, growing populations, climate change, worsening social inequality, and increasing noncommunicable diseases, the need for collaboration across urban planning and public health is crucial. While there are known harms in the urbanized environment, including air pollution and noise, there are also known benefits to human health related to urban form [5–7]. For example, built environment interventions that support active transportation, access to green space, and social connection have been shown to positively influence physical and mental health [8–12].

There are social and equity consequences of transportation planning decisions. As urban communities become increasingly car dependent and unaffordable, built environment interventions that support active transportation may increase mobility choices for all, but may specifically benefit populations without a car by enabling access to essential destinations such as work, healthcare, and school [13–15]. Yet evidence has shown that these groups experience inequitable access to active transportation infrastructure [16–19]. Thus, mitigating the inequitable distribution of active transportation infrastructure across neighbourhoods may result in improved access to opportunities for all residents and ultimately improved health. The COVID-19 pandemic required cities to rapidly implement built environment interventions to address new space and mobility needs. At the outset, there was little guidance and cities took different actions. In July 2020, the Federation of Canadian Municipalities released guidance for COVID-19 street reallocations [20]: temporary interventions that expanded street space for active transportation or physical distancing [21].

To understand what interventions work, where, and for whom, public health practitioners and researchers draw on a variety of tools and methods [22]. Population health intervention research, one such approach, allows researchers to test solutions and make recommendations for policy and practice-based actions [23]. To elucidate the relationship between health and equity, population health intervention research can generate knowledge about interventions at the population-level, considers systems of power, and the distribution of resources across population groups [22]. While evaluating intervention effectiveness in controlled, researcher-led settings is an important step towards generating evidence-based policy, investigating strategies of intervention implementation in real-world settings can strengthen the application of research evidence into policy and practice [24, 25].

Once intervention effectiveness has been established, public health practitioners and researchers need to understand how the intervention works in different contexts. Implementation science frameworks have been used across different timepoints in the research cycle (before, during, and after intervention implementation) and in various settings (clinical, community, and corporate). For evaluation, the RE-AIM (Reach, Effectiveness, Adoption, Implementation, and Maintenance) framework has been used to facilitate consistent reporting of implementation barriers and enablers [26]. In community settings, RE-AIM has been most commonly applied to evaluate public health projects, but our scan of the literature reveals a gap in application to built environment interventions [25, 27]. As public health expands beyond epidemiological studies and disease surveillance, attention towards the causes of community health is needed [25]. Thus, this study applies a social determinants of health lens to evaluate the implementation process and intended outcomes of a built environment intervention in urban settings using RE-AIM.

Using the RE-AIM framework, our aim is to evaluate COVID-19 street reallocation interventions in the cities of Vancouver (BC), Toronto (ON), and Montréal (QC), Canada. By applying RE-AIM to systematically evaluate the approaches of three Canadian urban areas, we can compare and contrast the barriers and enablers of implementation, and document lessons to take forward into future planning for active transportation interventions that hold implications for public health.

## Methods

### Geographical context

This study focused on three Canadian Census Subdivisions (CSDs) (~ municipality) of Vancouver, Toronto, and Montréal. Together, these municipalities make up 14% of Canada’s population. We purposefully selected these cities because their dense cores meant they had a pronounced need for physical distancing measures during the pandemic. Our study team has local expertise in each region. The spatial unit of analysis for this study is the census tract (CT) (population 2,500–8,000 [28]).

### RE-AIM framework

The RE-AIM framework, consisting of five domains of measures to evaluate public health promotions and interventions (Reach, Effectiveness, Adoption, Implementation, and Maintenance), was initially designed to help researchers consistently report results of evaluations of health promotion interventions [29]. Mixed method approaches are strongly encouraged to create a holistic picture of the implementation process [24, 30, 31]. To assess the RE-AIM dimensions, we draw on qualitative data from interviews with municipal staff responsible for street reallocations, and quantitative socio-spatial data (Table 1). These data were collected as part of a larger research program titled “Active Transportation Planning and Travel Behaviour Change in Post-COVID-19 Canada”, a Social Sciences and Humanities Research Council funded project.

**Table 1:** Assessment of RE-AIM indicators modified from [25, 30]

Domain	Built environment specific metric	Indicator (data source)
Reach	Estimate number of people reached based on the population living within a specific distance of the intervention	Proportion of the population living in CTs with a street reallocation (socio-spatial data)
		Proportion of the population belonging to a priority population living in a CT with a street reallocation (socio-spatial data)
Effectiveness	Assess intended goals and unanticipated consequences of the intervention	Accessibility (i.e., spatial proximity) of CTs with street reallocations to essential services (socio-spatial data)
Adoption	Assess the setting where interventions were implemented and the inclusion of community partners in approval of street reallocations	Description of pre-existing cycling infrastructure in CTs with street reallocations/Framework analysis of insights from people involved in decisions about intervention setting and implementation (spatial data/semi-structured interview data)
Implementation	Determine if and how streets were modified over time or if the reallocations were delivered as intended	Analysis of reflections about street reallocation intervention modifications over time or suggested future considerations (semi-structured interview data)
Maintenance	Assess plans for sustainability of street reallocation interventions over time	Framework analysis of insights relating to legacy of street reallocation interventions (semi-structured interview data)

Applying RE-AIM to built environment interventions in public spaces requires some adapting, in particular because the original indicators are healthcare and health outcome focused [25]. We made changes to RE-AIM terminology to reflect the urban planning context, for example, “target population”, “actor”, and “audience” was translated to “active transportation users”, “residents”, or “community members”. For this study, we align our evaluation of street reallocation outcomes to the intended goals of the intervention [25], and take a public health perspective. Further, in studies of built environment interventions we consider all residents as potential users of the intervention and examine the impacts on different priority population groups, instead of assessing outcomes as recommended in the original RE-AIM guidance (i.e. to calculate the proportion of people who participated in the intervention versus those who did not) [32]. For our study, we define priority population groups as youth (under age 15), older adults (age 65 and older), Indigenous people, people who identify as a visible minority (this is the term used in the context of the Canadian census referring to persons who are non-Caucasian in race or non-white in colour, excluding Indigenous peoples [33]), people who are Black, and low-income households.

These groups have been historically marginalized in Canada and have been burdened by discrimination, bias, and racism [34–38]. Priority population groups tend to live in areas with low accessibility to active transportation infrastructure [21, 39–42]. Therefore, these groups may benefit from improvements in access to active transportation infrastructure.

Finally, existing literature recommends a modification to the Adoption domain for built environment studies [25]. Adoption, in a healthcare context, assesses the extent to which the settings selected for the intervention are representative of settings that the users will visit. Some studies have described actors involved in approving the intervention as an indicator of Adoption [25, 27]. In considering the nature of transportation infrastructure, as networks and systems rather than isolated interventions, we decided to assess Adoption by describing both the pre-existing cycling infrastructure in the CT (i.e. the setting) and the people who were involved in decision-making.

#### Reach

This domain assesses the extent to which the intervention has reached the population. We evaluated the reach of street reallocations by calculating the proportion of the population who had access to street reallocation interventions. We then disaggregated the population into priority population groups as one way to assess if street reallocations reached those most in need of access to active transportation infrastructure. For each study city, we grouped CTs by quartiles for each priority population group (quartile 1 has the lowest proportion of people in that priority population; quartile 4 the highest proportion). We summarized the length of street reallocations in each CT and compared the distribution of street reallocations (by proportion) across the quartiles. Where a street reallocation coincided with a CT boundary, the length was attributed to both neighbouring CTs. To improve access for those who stand to benefit most from active transportation, CTs with the highest proportion of priority populations (Q4) should have received the greatest amount of street reallocation interventions.

#### Effectiveness

This domain considers the intervention’s intended outcomes. According to the Federation of Canadian Municipalities report, the purpose of street reallocations was to “provide residents with sufficient space to be physically active… [street reallocations] have been implemented to help address high pressure points on streets and sidewalks and enable safe mobility options near essential services in communities that rely more on walking and cycling” (p.5, FCM, 2020).

Accordingly, we use a measure of proximity to destinations (i.e., spatial accessibility to destinations) to evaluate whether street reallocations were located in areas with higher accessibility to essential destinations. The term accessibility is used in many ways in research and practice. For example, under the Americans with Disabilities Act, it refers to standards that ensure buildings or facilities are physically accessible for people with disabilities. Our use of the term accessibility aligns with transportation research, where it refers to the ease of reaching destinations via the transportation system. Given the intended purpose of street reallocations was to “enable safe mobility options near essential services” we look at accessibility to destinations, specifically those deemed essential during pandemic lockdowns: grocery stores, neighbourhood parks, healthcare, pharmacies, and employment. Our measure of accessibility does not reflect usage of street reallocations but provides an indication of whether the street reallocations were located in areas with greater potential access to these essential destinations.

#### Adoption

This domain describes the setting required for intervention uptake. We analyzed the CTs with street reallocation interventions in two ways. First, we assessed the pre-existing active transportation landscape in the CTs where street reallocations were located, to explore if street reallocations were implemented in areas that already had existing cycling infrastructure or not. Second, we used interview data to identify key people involved in the decision-making process and who had input on implementation (by providing support, approval, etc.) of street reallocations.

#### Implementation

This domain assesses intervention fidelity. We first described the nature of the street reallocations implemented in each city. Second, we used data from our interviews, where we asked participants about the evolution of the interventions in their city (e.g., changes or modifications that they made, and the rationale), and to reflect on lessons learned throughout the implementation process. Focusing on these adaptations allows an evaluation of whether the street reallocations were delivered as intended, or if they were modified over time, across settings.

#### Maintenance

This domain assesses sustainability of the intervention. We used interview data to understand how, two years on from initial implementation, municipalities have stewarded street reallocations.

### Data sources

We used quantitative and qualitative data sources to assess the RE-AIM domains, as described in this section.

#### Intervention

##### Street Reallocations

**Fig. 1:**
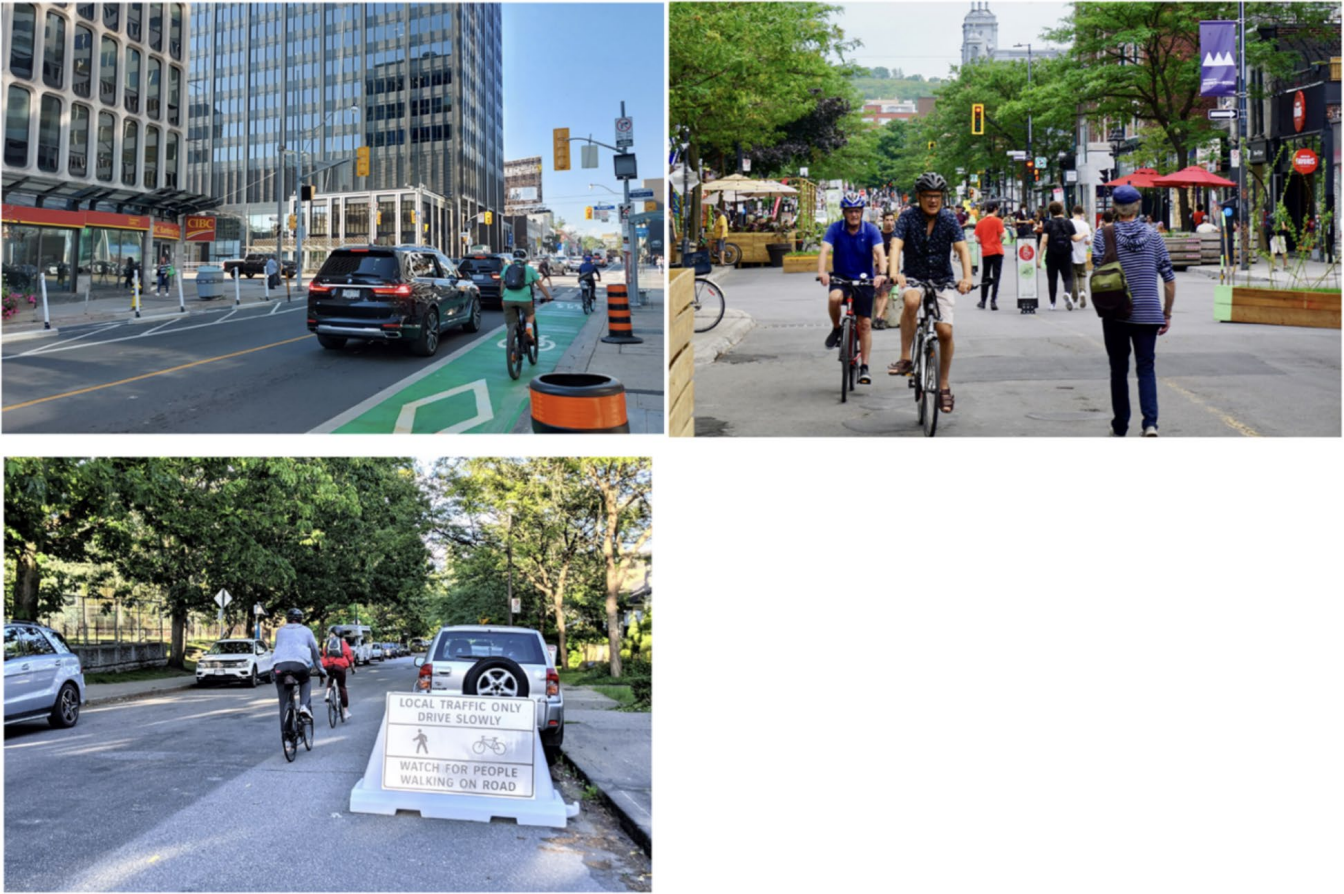
Examples of street reallocation types (clockwise): New bicycle lanes (Toronto, ON), Lane closures for pedestrians or cyclists (Montréal, QC), Quiet or slow streets (Vancouver, BC)

We included three types of street reallocations implemented between March-July 2020 [43]: new bicycle lanes or paths, lane closures for pedestrians or cyclists (including temporary week-long or weekend only closures), and quiet or slow streets that discourage private motor-vehicle traffic (e.g. Vancouver’s Slow Streets) as shown in Fig. 1. These types were grouped together for analysis. We sourced street reallocation locations from announcements on city websites and social media accounts.

Initial responses were notable because cities were under pressure to act quickly amidst public health uncertainty and changing mobility patterns (due to work-from-home orders) [44]. Decision-making was constrained by information availability, time, and resources compared to pre-pandemic time [45]. Responses therefore leveraged temporary infrastructure, such as bollards and signage, which were often readily available [46].

#### Socio-spatial data

##### Pre-existing cycling infrastructure

From the Open Data portals for each city, we downloaded the existing cycling infrastructure (built in 2019 or before), including protected bike lanes, local streets, painted lanes, and shared lanes (https://opendata.vancouver.ca/pages/home/; https://www.toronto.ca/city-government/data-research-maps/open-data/; https://donnees.montreal.ca/en/).

##### Area-level sociodemographic characteristics

With 2016 Population Census data available from Statistics Canada, we calculated the following, for each CT within our study area. We decided to use 2016 Census data because this would have been the demographic information available at the time of street reallocation responses:

1. Proportion of the population 65 years and older,
2. Proportion of the population under the age of 15,
3. Proportion of the population who are Indigenous,
4. Proportion of the population who are Black,
5. Proportion of the population who are Black,
6. Proportion of adults living below the after-tax low-income cut-off (LICO).

##### Proximity to destinations

To measure accessibility, defined as the extent to which individuals can reach essential destinations [47], we used the Proximity Measures Database (PMD, Statistics Canada, 2020). We focused on five services/amenities deemed ‘essential’ and that remained open during pandemic lockdowns: grocery stores, neighbourhood parks, healthcare, pharmacies, and employment. The PMD, created by Statistics Canada in 2020, provides information on proximity to 10 types of amenities based on a dissemination block centroid to centroid network distance. We aggregated this data to the CT-level and used the median score for analysis. The PMD uses a nationally normalized index value from 0 to 1 (lowest to highest proximity), so we created city-specific quartiles for our analysis.

#### Qualitative data

##### Semi-structured interviews

As part of the *﻿Active Transportation Planning and Travel Behaviour Change in Post-COVID-19 Canada* research program, we conducted a series of interviews with municipal practitioners who were involved in decisions about COVID-19 active transportation responses. Overall, 20 interviews were conducted across the Greater Metropolitan Areas of Vancouver, Toronto, and Montréal. For this study, we included content from the interviews in the cities of Vancouver (n = 2), Toronto (n = 4), and Montréal (n = 5). Our interview guide was based around the following themes: (1) the contemporary active transportation policy landscape; (2) the COVID-19 temporary street reallocation response; and (3) the evolution of street reallocation programming, including costs and benefits, maintenance, and lessons learned. The interview questions are included the supplementary materials of this manuscript. Interviews were between 45 min to 1 h in length and were conducted via Zoom in the spring and fall of 2022. Two interviewers were present for all interviews, and both conducted the coding of interview transcripts. Following the interview, personal identifiers were redacted from the transcripts. All transcripts, audio-recordings, and consent forms were saved with unique identifiers. We used NVivo 12 [48] for data management and content analysis.

Using framework analysis, we identified a thematic framework, and then indexed, charted, mapped, and interpreted the interview data [49, 50]. As an analytic technique, framework analysis is well suited to this study because it is geared towards policy and program-oriented inquiry [51]. Two coders read all transcripts and identified initial codes deductively (from a pre-established codebook) and inductively. For analysis, one coder organized related codes into themes. We selected quotes for inclusion in this analysis based on appropriateness to the theme, with an effort made to include quotes from across study regions.

## Results

Each study city implemented street reallocations to varying degrees. Overall, Toronto implemented the greatest length of street reallocations (114.6 km), followed by Montréal (102.1 km) and Vancouver (52.0 km; a smaller land area than other regions). In Toronto, street reallocations were mainly quiet or slow streets (56%), followed by new bikes lanes or paths (35%), and lane closures (9%). Montréal implemented mostly lane closures (83%), followed by new bicycle lanes and paths (11%), and quiet or slow streets (6%). Vancouver was notable for their Slow Streets program, which comprised 77% of their street reallocations, followed by lane closures (23%) and there were no new bicycle lanes or paths. Further study area characteristics are in Table 2.

**Table 2:** Description of study areas (2016 census) and street reallocation interventions implemented during March-July 2020

Variable	Vancouver (CSD)	Toronto (CSD)	Montréal (CSD)
Count of CTs	117	569	464
Land area (km^2^)	115.0	630.2	365.7
Population (2016)	631,486	2,731,571	1,704,694
Age characteristics			
0–14 years (%)	11.2	14.6	15.6
65 years and over (%)	15.5	15.6	16.0
Journey to work			
Walk mode share (%)	13.7	8.6	8.6
Bicycle mode share (%)	6.1	2.7	3.9
Transit mode share (%)	29.7	37.0	36.5
Sociodemographic characteristics			
Visible minority (%)	51.6	51.5	34.2
Black (%)	1.0	8.9	10.3
Indigenous (%)	2.2	0.9	0.7
Low-income status based on low-income cut offs, after tax (LICO-AT), (%)	18.0	17.8	20.2
Street reallocations (2020)			
Total length (km)	51.9	114.6	102.1
Length(km)/land area (km^2^)	0.4	0.2	0.3
Length(m)/Population (2016)	0.08	0.04	0.06
Existing bike network (2019)			
Total length (km)	340.3	755.8	823.1

### Reach

Street reallocation interventions reached between ~ 20–40% of residents: in Vancouver this was 39.9% of the population (*n* = 252,090/631,486); in Toronto, 21.0% of the population (*n* = 572,176/2,731,571); and in Montréal 32.9% (*n* = 560,844/704,694).

We found different patterning in terms of whether priority populations lived in areas where interventions were implemented (Fig. 1). In Vancouver, areas with more Indigenous people received more street reallocations (over 75% of street reallocations were in Q3/Q4 CTs), whereas areas with more visible minorities had less (only 33% of street reallocations were in Q3/Q4 CTs). A similar pattern emerged for Montreal. In Toronto, the distribution was more consistent, with closer to 25% of the street reallocations across each quartile of each socio-demographic group.

### Effectiveness

**Fig. 2:**
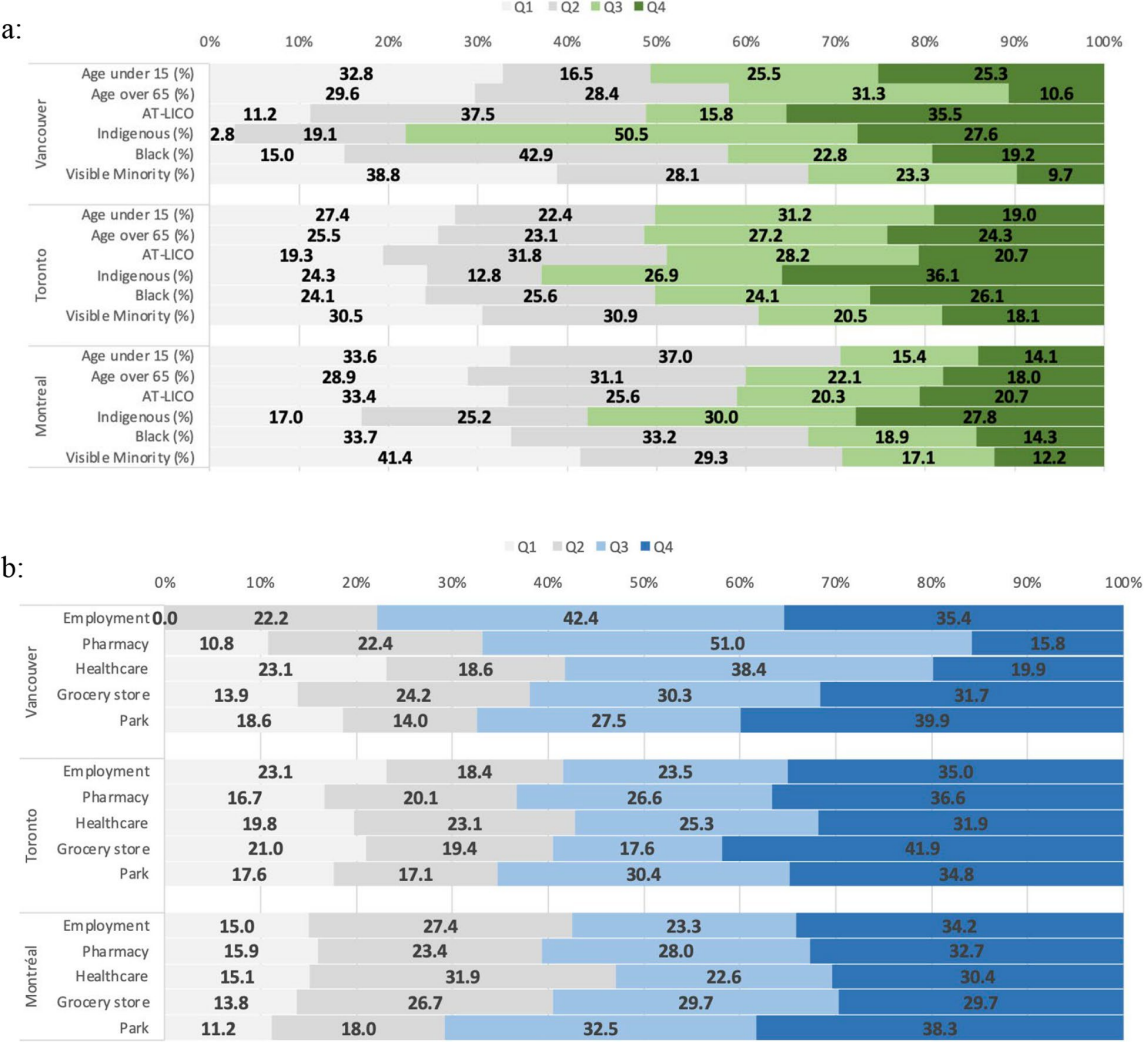
**a** Percent of total street reallocations by quartile for socio-demographic measures, by city (CSD). **b** Percent of total street reallocations by quartile for accessibility measures, by city (CSD)

The effectiveness domain examined accessibility to destinations, specifically employment, pharmacy, healthcare, grocery stores, and parks. Our analysis of accessibility data demonstrates that street reallocations achieved their intended purpose of facilitating spatial accessibility to essential services (Fig. 2). Across all cities, the highest proportions of street reallocations were implemented in CTs with high accessibility (Q3 or Q4) to essential services. In Vancouver, CTs with high accessibility to employment received 77.8% of street reallocations (Q3 and Q4). In Montréal and Toronto, 70.8% and 65.3% of street reallocations were in CTs with high accessibility to parks, respectively (Q3 and Q4).

### Adoption

**Fig. 3:**
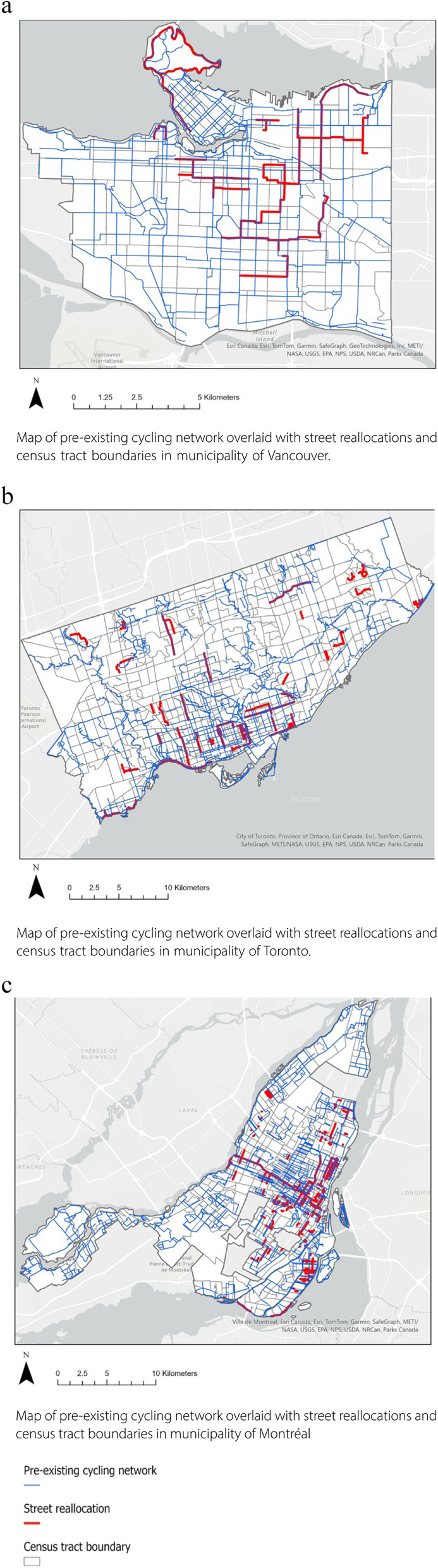
**a** Pre-existing cycling network and street reallocations, Vancouver (CSD). **b** Pre-existing cycling network and street reallocations, Toronto (CSD). **c** Pre-existing cycling network and street reallocations, Montréal (CSD)

The quantitative analysis for the adoption domain looked at if street reallocations measures tended to be implemented in areas that already had cycling infrastructure (i.e. further developing the pre-existing cycling network) or not. Figure 3a, b, c displays maps of Vancouver, Toronto, and Montréal visualizing where the pre-existing cycling networks and street reallocations were located in each city.

In Vancouver, all CTs where street reallocations were implemented had cycling infrastructure before 2020 (n = 117). In Montreal, only 9/464 CTs where street reallocations were implemented did not have pre-existing cycling infrastructure and in Toronto, this was 24/569 CTs.

Adoption of built environment interventions in municipal settings depends on members of the community. The community members who were influential in decision-making, according to interview participants, were the mayor, members of council, residents and local Business Improvement Associations (BIAs). Across all interviews, participants spoke to the importance of aligning street reallocation interventions with nearby residents’ needs, as the intended users of the infrastructure. When consultation with residents was not possible (due to expedited timelines or pandemic restrictions) municipal staff involved council or local councillors to speak as proxies for the residents. Though not the primary intended users of the infrastructure, local businesses were also consulted regarding the location of street reallocation implementation. In Toronto, a local BIA did not support a particular street reallocation intervention, which resulted in the project being implemented elsewhere the following year:

*We consulted with the Business Improvement Association that represented the businesses along that road corridor for the road closure project… [they] weren’t very supportive because they felt that with the pandemic the businesses were already suffering… so that’s why, after that first year, we moved the location to a different street*.

In contrast, in Vancouver, participants recognized limitations of doing engagement via BIAs, in that while this was not a new process, the pandemic put a spotlight on parts of the city that are not represented by commercial partners and thus may not have had a voice in decision-making, *“There are parts of the city that don’t have BIAs and it was harder for them to connect in with the city and kind of bring their ideas forward”*. Similarly, participants in Montréal recognized limitations in their engagement with businesses, but for a different reason. Municipal practitioners spoke to finding a balance between priorities of all community partners involved in street reallocation implementation:

*It is important to document and always listen to the population of the business community and to show them that we have this objective of working with them. But at the same time, we must not forget our objectives; our objectives in sustainable development, in reducing greenhouse gas emissions… we must be ready to make changes that will not please everyone*.

As such, a barrier to implementation may have been balancing community priorities with municipal objectives. While street reallocations were implemented during a time of public health concern, the participant notably did not reference health-related goals. Instead, the focus on sustainability and reducing emissions underscores the broader policy aims driving these interventions. Street reallocations affected a wide range of community members and had the potential to support diverse policy objectives beyond their original scope.

### Implementation

In each city, we heard that street reallocations were implemented and then adapted. Interview participants shared that some of the best lessons to take forward about implementation came from situations where street reallocations were removed. Street reallocation removals resulted from iterative feedback cycles with residents or other community partners, and municipal practitioners learned to develop clear communication strategies. As a participant in Montréal expressed, one such communication strategy may not include robust consultation; *“The fact of communicating better with citizens, maybe not always consulting because everyone wants to be consulted on everything, but having a communication strategy”.* In many instances, these interventions were implemented as pilot projects to allow users to experience the street reallocation before providing feedback. This quote speaks to this important shift, where practitioners learned that consultation before implementation is not always effective. By keeping lines of communication open, a participant in Vancouver expressed similar ability to be responsive:

*In, like, 6 out of 7 cases the neighbourhood came back and was like ‘that thing was great’ and in one case the neighbourhood was like ‘yeah, we don’t really like that so much’, so we got out there really quickly and changed that*.

Similarly, in Toronto, street reallocations were designed to be iterative, and a participant spoke to adapting the interventions according to feedback; “*and if we would get complaints, routes were tweaked a bit based on public feedback”.* On-going feedback and communication with community members therefore facilitated street reallocation adaptations and may have enabled implementation.

### Maintenance

In terms of Maintenance, our interviews provided an understanding of maintenance and sustainability of street reallocations during the pandemic only. Practitioners expressed difficulty in predicting long-term sustainability due to changing priorities in the city. In Montréal, a participant shared “*[The street reallocation] duration and length, probably, will vary over the years according to the wishes of the merchants*” in relation to a particular intervention that was pedestrianized during the pandemic, highlighting the uncertainty of the coming years. Similarly, a participant in Toronto expressed the importance of the pandemic for generating support; *“I think it's a ‘How can we sustain the support…the unprecedented support that this portfolio got for this very brief time, right?’”.* The participant went on to reflect that the question of maintenance of street reallocations is actually representative of a need for ongoing support for active transportation infrastructure.

The temporary materials used were also a discussion point related to maintenance. In Vancouver, practitioners discussed the difficulty and costliness of maintaining temporary street reallocations because the materials blew away in the wind and were easily moveable. In Toronto, we heard a similar reflection, *“[Street reallocations] went in with, like, what we call the quick build materials or the modular materials, and those break down faster, or get hit or shifted by people”.* The participant reflected on implications for staff capacity. The city departments involved in road works and maintenance had to adjust quickly to a new pace of implementation regularly factor in upkeep.

## Discussion

We used the RE-AIM framework to evaluate street reallocation implementation in the cities of Vancouver, Toronto, and Montréal. Our analysis found several enablers (i.e. factors that facilitated intervention uptake) and barriers (i.e. factors that led to intervention removal) that can be taken forward to inform future active transportation planning with implications on public health. Enablers for street reallocation implementation included clear and on-going communication, and iterative feedback cycles. In terms of barriers, while instrumental for rapid implementation of street reallocations, the temporary materials used required considerable upkeep and were unsustainable strains on municipal resources.

### Public health impacts of street reallocations

Although the street reallocations analyzed in this study were COVID-19 pandemic responses, they can be seen as fitting within a broader movement of tactical urbanism and experimental street transformations [52]. The COVID-19 pandemic experience may have strengthened the practice of using temporary installations as a precursor to permanent built environment interventions with the capacity to impact population health. With auxiliary benefits, such as promoting physical activity, healthy food access, and social connectedness for nearby residents, street reallocations can potentially serve as tools for advancing public health goals. Our analysis cannot confirm if street reallocations led to an increased use or changes in travel behaviour.

We used accessibility (i.e., spatial proximity to destinations) to measure effectiveness of the interventions, but without usage data, we cannot assess the extent to which reallocations improved health or influenced mobility. Future research should integrate individual-level mobility data or travel diaries to assess behavioural outcomes directly. Studies using survey and mobility data have started to provide evidence that street reallocations were associated with walking and cycling [53, 54], demonstrating the health-related impacts of these interventions.

### Equity implications of street reallocations

Major questions have arisen about the equity impacts of COVID-19 planning decisions [37, 46, 55–57]. Our research provides some insights on the accessibility of street reallocations across population groups using an objective way of characterizing socio-spatial equity. We found that in Vancouver and Montréal, areas where more Indigenous people live received more street reallocations, whereas areas where more people who are visible minorities live received fewer interventions. Rather than attributing this finding to an explicit intention of decision-makers, we recognize that pre-existing land use, settlement patterns, and locations of ethnic enclaves in urbanized areas may impact access to active transportation infrastructure. Over time, new infrastructure implemented following these same patterns may exacerbate inequities; a concern that has been raised by studies focused on the Toronto area [58, 59]. In Toronto, some street reallocations expanded to the historically underserved eastern and northern parts of the city. Limited connections to the existing infrastructure, however, diminished accessibility gains [58]. In contrast, the street reallocation implementation in the central urban core, where amenities were more concentrated and connections with pre-existing infrastructure were strengthened [58].

Research from across the globe asserts that pre-existing municipal transportation plans and sustainability goals influenced pandemic decision-making [57, 60]. As equity is increasingly a focus of urban planning and public health discourse, this is a space for future development. Our findings, that street reallocations were implemented in areas with high amenity density and pre-existing cycling infrastructure, signal that communities who stand to benefit most from new access to active transportation infrastructure may not have gained street reallocations. While Toronto and Montréal implemented some street reallocations in underserved areas, these were limited in scope. Thus, overall, the interventions may have reinforced existing inequities, rather than mitigating them.

The impacts of street reallocations on commuter mobility require further study from a public health perspective. Access to employment, a social determinant of health, affects an individual’s ability to live a full and healthy life. Concerns have been raised that street reallocations may have posed challenges for essential workers commuting during the pandemic, so examining this impact in the long-term is needed [61]. Based on research on pre-pandemic street reallocations, initial fears of mobility disruptions are often overestimated [62]. The public tends to adapt to changing street conditions over time, finding alternatives as needed [62, 63]. This adaptability highlights the long-term potential of street reallocations, with impacts that may become increasingly apparent as residents adjust.

While we acknowledge that RE-AIM does not explicitly focus on equity, implementation science can contribute to advancing equity goals [64, 65]. Our study, using a reproducible method to evaluate equitable accessibility of street reallocations across sociodemographic groups, contributes to this growing body of equity measures for built environment interventions [44]. In future studies of street reallocations, particular attention could be on the impacts to non-car users, because pandemic-time studies highlighted burdens on pandemic commuters, namely younger, lower-income, and for minority groups [66]. These groups often face systemic barriers to mobility and accessibility, and it is unclear if new active transportation infrastructure reduces inequities or inadvertently reinforces them [67].

### Lessons for future research and practice

Our findings contribute to the public health and transportation literature by evaluating the implementation process of an intervention under rapid decision-making timelines. Many studies have acknowledged the urgent need presented by the COVID-19 pandemic to act on broader health and environmental goals across cities [68–71]. In the pandemic urgency, municipalities trialed new ways of implementing infrastructure, including experimental designs with temporary materials and expedited or omission of public consultation processes [57]. While there are known concerns about the top-down decision making exhibited during pandemic active transportation planning [72], our findings illustrate that open feedback cycles may have enabled municipalities to collect data about community relevant priorities, leading to uptake and acceptance of street reallocations in some instances.

Intervention adaptations were important to ensure interventions continued to meet residents’ needs over time. While practitioners noted that the use of temporary materials was costly to maintain, these materials may have ultimately contributed to cost savings by allowing for easier removal or modification in response to the community feedback that led to street reallocation adaptations. Researchers and practitioners may evaluate this adaptation and continuous engagement approach in future work, for example by using an equity-centred evaluation framework such as the Adapted Proctor Framework [73, 74]. Our study highlights three additional gaps: the need for (1) disaggregated data collection, (2) monitoring of behavioural outcomes, such as usage, and (3) targeted consultation with identified priority populations early in implementation.

### Limitations

This research has limitations. Our study was not initially conceptualized using an implementation science framework, and so we are limited by the data available at the time of analysis. A typical indicator of Effectiveness in RE-AIM studies is usage of the intervention. Our team did not collect data on usage; in fact, few cities had plans or resources to reliably collect this data in the early years of the pandemic. Further, we note there is no explicit domain for equity within RE-AIM, despite the increasing focus on equity in both health and planning. We conducted interviews with municipal practitioners two years following the initial onset of the pandemic. Employee turnover and changes following the pandemic may have limited our ability to reach relevant staff. While we attempted to reach all potential participants through our sampling, our semi-structured interviews may not reflect the opinions of all decision-makers involved in street reallocation implementation.

## Conclusion

This study contributes to the public health literature by evaluating the real-world implementation of street reallocations, interventions that have the potential to improve population health outcomes. Our analysis using the RE-AIM framework, resulted in an understanding of factors that influenced the barriers (e.g., material constraints) and enablers (e.g., iterative feedback) of street reallocation implementation in different Canadian contexts. As we move forward using RE-AIM to evaluate built environment interventions, more attention is needed to incorporate consistent measures, including usage, health, and equity metrics, that align with urban planning, health, and the intersection of these disciplines. In practice, this means adopting structured evaluation frameworks, collecting disaggregated data, and maintaining continuous engagement to ensure interventions address community needs in both emergency and non-emergency contexts.

## Supplementary Information

Supplementary Material 1

## Data Availability

All datasets used an/or analyzed during the current study are available from the corresponding author on reasonable request.

## Abbreviations

RE-AIM: Reach, Effectiveness, Adoption, Implementation, Maintenance
CT: Census tract
AT-LICO: After-tax low income cut off
PMD: Proximity measures database
