# Supplementary material for: Evaluating the implementation of active transportation infrastructure during the pandemic: a RE-AIM study
Source: BMC Public Health. 2025 Dec 20;26:874. doi: 10.1186/s12889-025-25432-y (PMC12980895; doi:10.1186/s12889-025-25432-y)
Supplement: Supplementary file 1 — Supplementary Material 1 [file 12889_2025_25432_MOESM1_ESM.docx]

**INTERVIEW GUIDE**

CONTEXT AND BACKGROUND:

1. As a Transportation Planner in the City of X, what was it like during the pandemic? What initiatives were you involved in?
2. What are the different departments/sections within your municipality that are involved in active transportation planning in [City]?

MOTIVATIONS OF TEMPORARY STREET REALLOCATION PLANNING:

1. Thinking back to when the decision to implement pandemic-time street reallocations first occurred in your municipality, were there notable changes in priorities from pre-pandemic times that would have influenced how these decisions were made?
2. Do you think pandemic-time priorities are still relevant to planning today and will they keep their relevance in the future?

DECISION-MAKING PROCESS:

1. Can you describe the decision-making process behind the street reallocation initiatives during COVID-19?
2. Was community engagement conducted?
3. In designing the pandemic-time street reallocations, did you consider equity?
4. Have the existing Planning instruments (such as, official plans, transportation plans, or environmental plans) helped or hindered the rollout of pandemic-time street reallocation projects? In what way?
5. Did municipal action plans for pedestrian and cyclists’ safety factor into the decisions made of where and how to reallocate street space? (For example: Montreal & Toronto: Vision Zero Action Plan, Vancouver: Moving Towards Zero Safety Action Plan, Transport 2040 Plan, etc.)

EVALUATION AND FUTURE IMPACTS:

1. How are the street reallocation projects being evaluated?
2. What lessons have been learned for future active transportation planning in the City of X?
3. Any final thoughts that you wanted to share about COVID-19 street reallocations or the future of active transportation planning in [City]?
4. Is there anyone else you think we should speak to in [City]?
